# Analysis of Multiscale Condensation Phenomena Using a Zero‐Shot Computer Vision Framework

**DOI:** 10.1002/advs.202521372

**Published:** 2026-01-07

**Authors:** Donghyeong Lee, Seokwan Roh, Jaewoo Jeong, Kuk‐Jin Yoon, Jungchul Lee, Youngsuk Nam

**Affiliations:** ^1^ Department of Mechanical Engineering Korea Advanced Institute of Science and Technology (KAIST) Daejeon Republic of Korea

**Keywords:** computer vision, droplet condensation, phase‐change phenomena

## Abstract

Understanding and controlling condensation is central to diverse energy and water systems, yet quantifying its inherently multiscale droplet dynamics has remained elusive. Here, a label‐free computer vision framework that leverages the Segment Anything Model (SAM) for zero‐shot segmentation is demonstrated, achieving high accuracy by systematically detecting more than one million droplets without any annotated datasets or retraining. The framework extracted statistical features such as droplet radius, number of coalescences, mean coalescing diameter, growth rate, and condensation mass from microscale to macroscale observations. This enabled direct visualization of dynamic condensation cycle including nucleation, growth, coalescence, sweeping, and renucleation, while also revealing the role of surface properties, where contact angle hysteresis governed droplet departure size, morphology, and population statistics in agreement with classical models. To extend beyond characterization, a machine learning model was trained with Pearson correlation–selected parameters to predict condensation rates under diverse environmental conditions, achieving a mean absolute percentage error of 7.8%. These frameworks highlight the potential of artificial intelligence to understand dynamic phase‐change mechanisms, and to guide the design of advanced surfaces and systems for thermal management, desalination, and water harvesting.

## Graphical abstract

A zero‐shot computer vision framework quantifies multiscale condensation dynamics by automatically segmenting droplets and extracting physical parameters without labeled data. The workflow integrates data mining and statistical analysis to reveal droplet growth, coalescence statistics, and sweeping behaviors, enabling label‐free measurement of heat flux and condensation mass through artificial intelligence–assisted analysis.

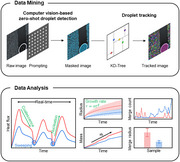

## 1 Introduction

Condensation enables rapid heat and mass transfer via phase change. Therefore, this phenomenon plays a crucial role in various industries, including power plants [1], heating, ventilation, and air conditioning (HVAC) [2], desalination [3], and water harvesting [4]. Among the two primary condensation modes, dropwise condensation forms discrete droplets on non‐wetting surfaces and achieves significantly greater heat transfer than filmwise condensation, which involves a continuous liquid film with high thermal resistance [5, 6]. In particular, dropwise condensation involves a dynamic sequence of nucleation, growth, coalescence, and removal by gravitational sweeping, which continuously refreshes the surface and facilitates efficient heat exchange. A comprehensive understanding of how this droplet behavior is influenced by environmental parameters such as supersaturation level, relative humidity, and surface temperature is essential for optimizing the performance and efficiency of dropwise condensation systems [7]. Despite the importance of this process, its mechanisms remain ambiguous owing to the stochasticity and inherent complexity of droplet behavior.

As heat and mass transfer performance is significantly governed by the behavior of droplet interactions such as droplet‐droplet and droplet‐surface, numerous recent studies have attempted to quantify the dynamics of condensation. Based on classical phase‐change theory, these studies have used image‐based techniques (e.g., ImageJ) to extract droplet‐level dynamics from experimental images [8, 9, 10]. However, in most of these studies, droplet‐level information is extracted using user‐defined thresholding and rule‐based image‐processing workflows, which require substantial human intervention and tend to be unstable and labor‐intensive when applied to large datasets. Moreover, quantifying millions of droplet interactions in real‐time remains challenging due to limitations of the temporal and spatial resolution of imaging systems, as well as the complexity of condensation phenomena including multi‐droplet coalescence and gravity‐driven sweeping.

To address this issue, deep learning‐based computer vision techniques have been increasingly employed in phase‐change image segmentation [11, 12, 13, 14, 15, 16, 17, 18, 19]. These methods enable the quantification of thousands of droplets by generating segmentation masks on actual droplets, thereby facilitating the analysis of dynamic droplet behavior. Suh et al. developed a vision‐based framework that employs a Mask R‐CNN model trained on labeled images including thousands of droplet images [20]. This framework enables automated extraction of key physical features, such as droplet size and location, thereby analyzing the critical information including heat flux and droplet number density. Ma et al. used a custom YOLO‐v4 model and adopted data augmentation techniques, including mix‐up and copy‐and‐paste, which expanded the limited set of human‐labeled images to a dataset of more than 10 000 annotated images [21]. Using this model, they observed droplet coalescence and jumping behavior on a superhydrophobic lattice‐structured surface, which enabled the identification of the optimal cut‐off size for droplet removal and the subsequent optimization of the lattice design.

However, collecting labeling data of computer vision models for training or fine‐tuning remains a significant challenge. This is because the training process of the model requires a large amount of labeling data, which has substantial human‐labor and cost [22, 23, 24]. Moreover, conventional CNN models are no longer applicable to new tasks that exhibit different droplet morphologies caused by varying substrates, lighting, and orientations, which highlights a key limitation in their generalization. Another limitation is the assumption that droplets are perfectly spherical, whereas the actual droplet can become pinned caused by chemical and structural heterogeneities on the surface, leading to increased contact angle hysteresis [25, 26, 27]. This discrepancy can result in a mismatch between the vision‐based analysis and the actual physical behavior of droplet motion. Therefore, it is essential to accurately consider the actual droplet morphology in analysis. In addition, most previous studies have primarily focused on droplets smaller than tens of micrometers, which are typically observed using high‐magnification microscopes [20, 24, 27]. Hence, sweeping phenomena involving droplets larger than the departure diameter have been rarely investigated. Furthermore, many classical condensation heat transfer models have used a mean coalescing radius (e.g., *r*_e_) [5, 28]. However, this parameter is difficult to obtain experimentally, because coalescence events occur stochastically and highly depend on surface characteristics. Therefore, determining the mean coalescing radius from experimental data remains a significant challenge.

In this study, we present a vision framework that does not require additional model training or fine‐tuning by leveraging a vision‐transformer (ViT) model. We employed the Segment Anything Model (SAM) developed by Meta AI, which is a ViT‐based foundation model [29]. SAM has been pre‐trained on a large dataset, thereby enabling zero‐shot generalization for instance segmentation, which allows the model to segment previously unseen objects without any task‐specific training or annotations. To adapt the model to our specific task, we adjusted the parameters of the model such as the non‐maximum suppression (NMS) threshold and the number of point per batch without using any labeled images. As a result, this framework enables real‐time monitoring of thousands of droplets on silane‐coated silicon and polycarbonate surface with robust detection even for deformed droplets resulting from high contact angle hysteresis. By leveraging our vision framework, we analyzed more than one million droplets to extract condensation statistics including eccentricity, condensation mass, heat flux, droplet size distribution, and growth rate. In addition, our framework can quantify the number of coalescence events and the mean coalescing radius. Furthermore, a surrogate model based on a machine learning approach was developed to predict condensation rates for various conditions, thereby revealing key factors such as supersaturation level, relative humidity, and surface temperature. Overall, our research suggests an innovative vision‐based approach for condensation analysis without the need for labeled images, bridging the gap between traditional phase‐change theory and data‐driven statistical methods.

## 2 Results and Discussion

### 2.1 Workflow for Computer Vision‐Based Analysis of Condensation Phenomena

**FIGURE 1:**
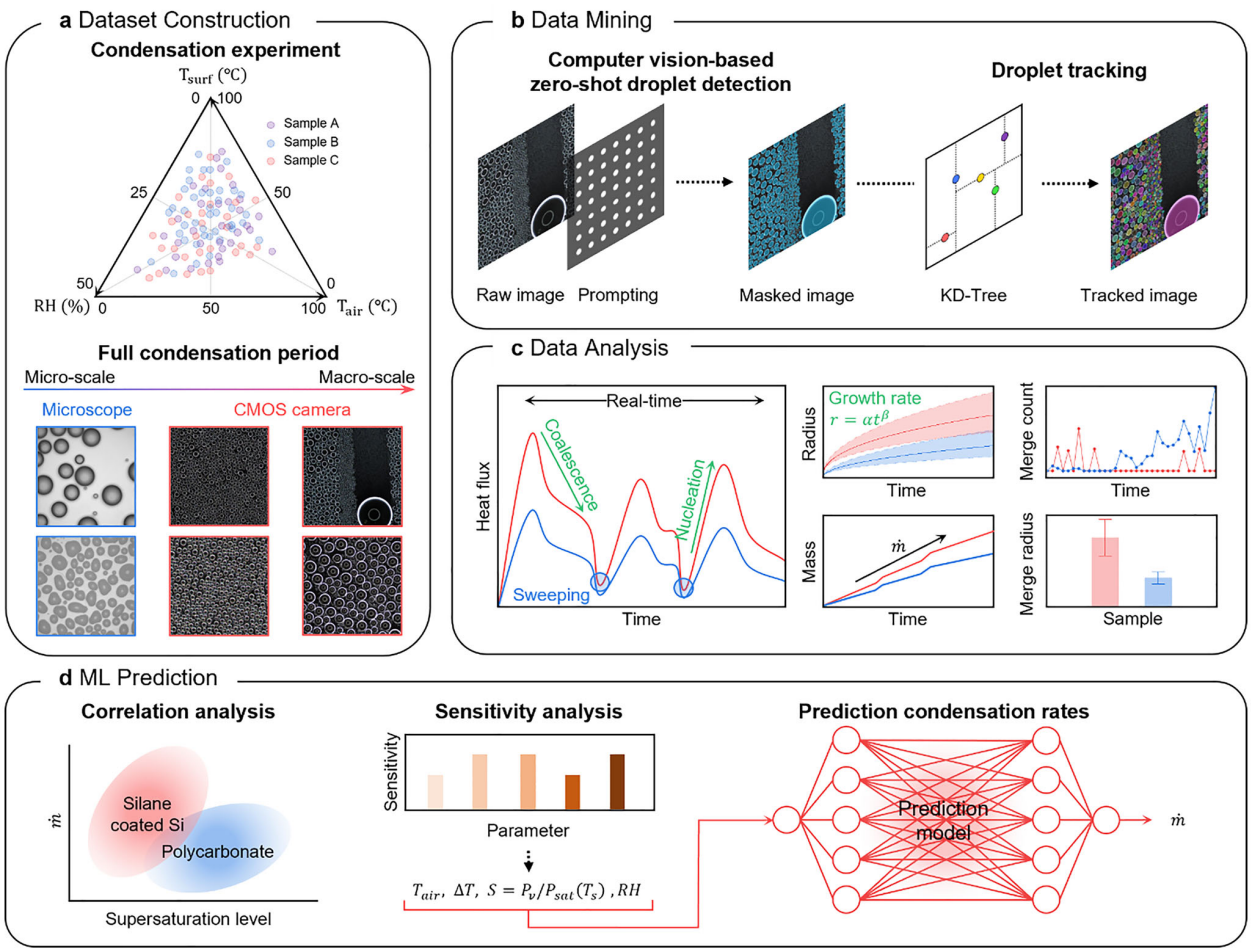
Workflow of the computer vision‐based analysis of condensation phenomena. (a) In the dataset construction stage, condensation images are obtained under various environmental conditions by controlling relative humidity, surface temperature, and air temperature. (b) In the data mining stage, these images are then processed through a computer vision model to detect droplets, resulting in a masked image. The mask (blue) represents the generated mask on the droplets, which includes data such as droplet radius, droplet count, and positions. (c) In the data analysis stage, we extracted meaningful data including heat flux, droplet growth rate, condensation mass, and coalescence frequency. (d) In the machine learning prediction stage, correlation analysis is first conducted to identify the most relevant input parameters. The trained model was then developed using the condensation rate and the selected condition data. This model directly predicts the condensation rate for untested environmental conditions based on the given inputs.

We developed a model framework consisting of dataset construction, data mining, data analysis, and machine learning prediction (Figure 1). Figure 1a illustrates the dataset construction stage, where condensation experiments by controlling relative humidity, ambient temperature, and surface temperature on various samples are conducted to build an image dataset. Next, the data mining stage (Figure 1b) processes these images by inputting them into a computer vision model. The outputs of the model are masks rendered in blue, assigning a droplet‐like color for easier recognition. These masks include the physical features of individual droplets, such as droplet area, radius, the number of droplets, and location. These outputs are stored in a dataframe and used for statistical analysis. Next, we conducted droplet tracking. Individual droplets are tracked using a *k*‐dimensional tree algorithm, which is a spatial partitioning method for organizing droplet positions on the samples [30]. In this step, spatial features of individual droplets, such as location, radius, and eccentricity are utilized as parameters to assign a unique ID. Consequently, as time steps progress, droplets are linked to their prior time step unique ID, allowing the tracking of their physical feature changes over time. Figure 1c shows the data analysis stage, where the physical features of droplets obtained from the prior step are statistically analyzed and quantified to convert to meaningful features, such as condensation mass, heat flux, and growth rate. This stage focuses on the analysis of the dynamic behavior of condensation droplets, including droplet nucleation, growth, coalescence with neighboring droplets, and sweeping by gravitational force. Figure 1d shows the machine learning prediction stage. At this stage, a model was developed to predict the condensation rate under specific environmental conditions, based on the data obtained in the preceding steps. Before the beginning of machine learning modeling, Pearson correlation analysis was used to determine the model input parameters to identify the key factors highly correlated with condensation performance. Then, the surrogate model is developed to predict condensation rates and heat flux under various environmental conditions. Overall, our model framework provides a quantitative understanding of the condensation process by linking experimental data, droplet‐level physical analysis, and data‐driven prediction under varying environmental conditions.

### 2.2 Computer Vision Model

**FIGURE 2:**
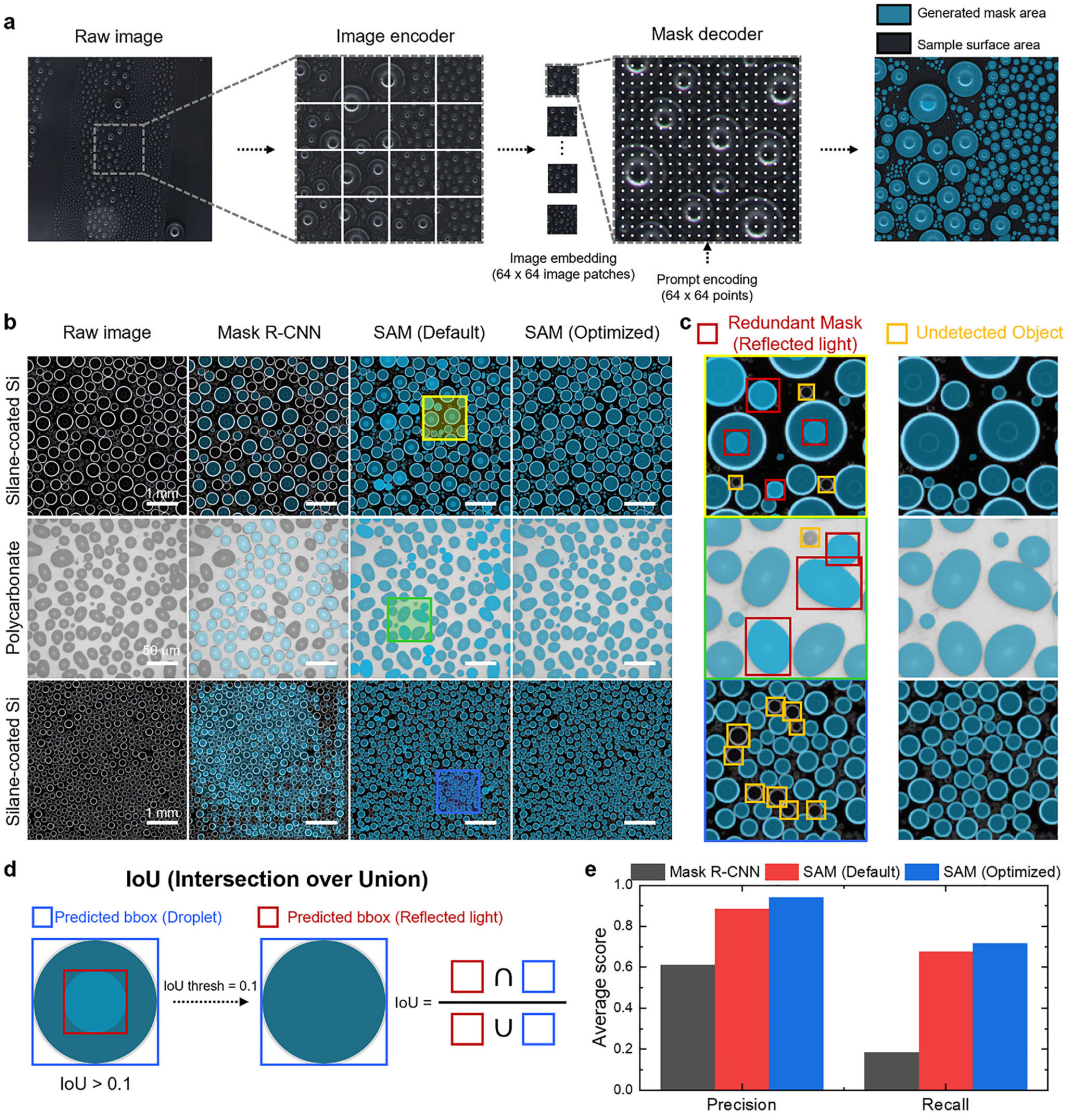
Zero‐shot segmentation of condensation droplets using SAM. (a) Workflow of the vision transformer–based SAM for droplet segmentation. The raw image (2048 × 2048 pixels) is cropped to 512 × 512 pixels to fit the interest observation scales. This cropped image is then subdivided into 16 downscaled image patches, then embedded. Image patch is prompted with 64 × 64 regularized points. Using these points, the location of the droplets is extracted, and a droplet mask is generated and overlaid onto the original image. (b) Comparison of segmentation results on different surfaces using Mask R‐CNN, default SAM, and optimized SAM with a scale bar of 1 mm and 50 µm for silane‐coated Si and polycarbonate, respectively. (c) Magnified regions from (b) highlighting segmentation errors in the default SAM, including redundant masks from reflected light (red) and undetected small droplets (yellow), which are mitigated by optimization. (d) Non‐maximum suppression (NMS) refinement to remove redundant masks, illustrated with bounding boxes and IoU thresholding. (e) Quantitative evaluation of segmentation performance showing that the optimized SAM achieves the highest precision (0.94) and improved recall (0.72) compared to default SAM and Mask R‐CNN.

Our study aims to segment droplets from various samples without training or fine‐tuning. Our model is a modified Segment Anything Model (SAM), a vision transformer–based foundation model pre‐trained on a large‐scale dataset [29, 31]. Owing to its zero‐shot generalization capability, SAM can recognize and segment previously unseen objects without task‐specific training. The mask quality of this model is greater than 90% IoU (Intersection over Union). The model checkpoint was based on ViT‐H, pre‐trained on the SA‐1B dataset, which contains over 1.1B masks from 11 m images across various domains [32]. Figure 2a illustrates the workflow of the computer vision model that processes experimental images to generate mask images. The SAM architecture consists of an image encoder, a prompt encoder, and a mask decoder. Before being processed by the model, the 2048 × 2048 pixel images obtained from the condensation experiments are cropped to the central region of 512 × 512 pixels. To ensure that the condensation amount is not biased by the choice of image region, we performed a random region crop test in Figure S2 and Note S2. When the image is input into the model, the output of the image encoder is a 16× downscaled image embedding. The prompt encoder identifies the specific regions of the image that require segmentation of droplets. The point prompt consisting of a 64 × 64 regular grid of points was used in the image. The mask decoder maps the image embedding and a set of prompt embeddings to an output mask. The generated mask image is then upscaled to the original size. This process allows the extraction of physical information including droplet area, equivalent radius, the number of droplets, and droplet location for thousands of individual droplets per image, by converting pixel‐based measurements in the segmentation mask to physical units using the calibrated pixel size (Experimental Section). All SAM‐based segmentation and droplet‐feature extraction were performed on a workstation equipped with an NVIDIA GeForce RTX 3090 GPU. On this system, processing a single 512 × 512 pixels condensation frame from the input to mask generation takes approximately 30 s.

To enhance droplet segmentation performance, we optimized the options of the automatic mask generator at the inference stage while keeping all SAM weights fixed. SAM is a generalization model trained on diverse object classes, including natural scenes, humans, and everyday objects achieving high average precision (AP) on general photographic datasets. Figure 2b shows segmentation results from Mask R‐CNN, default SAM, and optimized SAM across different surfaces, highlighting specific areas magnified in Figure 2c. However, when SAM is applied to condensation images containing thousands of droplets, reflected light on droplet surfaces can lead to redundant masks (red boxes) causing overestimation, or failure to detect extremely small droplets (yellow boxes), as shown in Figure 2c. To minimize such errors, we adjusted only inference‐stage hyperparameters of the SAM automatic mask generator—such as the non‐maximum suppression (NMS) threshold, the number of points per side and per batch, and the minimum/maximum mask area—without using any labeled condensation images, so that the pre‐trained SAM weights remained unchanged while the configuration was adapted to high‐density droplet environments specific to condensation experiments. To further verify the generalizability of this optimization in the condensation domain, we show that our method remains qualitatively robust across diverse substrates, including bare Cu, Al, Fe, and Ti as well as glass, and also identifies droplets in out‐of‐focus images originating from height variations on a nanostructured surface (see Figure S6).

Figure 2d illustrates the application of box NMS threshold to refine droplet segmentation results obtained from SAM. In the figure, the blue bounding box indicates a correctly segmented droplet mask, while the red bounding box represents a redundant mask due to reflected light on the droplet surface. Intersection over union (IoU) quantifies the overlap between these bounding boxes and is defined as:

(1) $$\mathrm{IoU}\;=\frac{Area\left(\mathrm{Red}\cap \mathrm{Blue}\right)}{Area\left(\mathrm{Red}\cup \mathrm{Blue}\right)}$$

For instance, setting an IoU threshold of 0.1 removes redundant masks by suppressing overlapping masks with IoU values greater than 0.1. Consequently, only the bounding box corresponding to the actual droplet (blue box) remains, effectively mitigating redundant masks caused by reflected light. This optimization significantly improves segmentation accuracy in high‐density droplet environments (see Figure S3)

To compare the performance of models, we evaluated the optimized SAM against both the default SAM and Mask R‐CNN, a widely used baseline in the field of the phase‐change heat transfer field (Figure 2e). [15, 17, 18] Mask R‐CNN employed a ResNet‐based backbone pretrained on the MS COCO dataset, while SAM utilized a ViT‐H/16 encoder pretrained on the SA‐1B dataset through a self‐supervised method (Masked Autoencoder, MAE). All models were evaluated without fine‐tuning. Evaluation was performed on 50 condensation images containing 23 000 manually annotated droplets under various environmental conditions (details in Note S3). Mask R‐CNN achieved the lowest precision of 0.61 because it was not trained on droplet condensation images, leading to pronounced domain shift. In contrast, SAM demonstrated strong zero‑shot generalization, with the default SAM achieving a precision of 0.89. However, the default SAM often produced redundant masks by misidentifying reflected light as droplets. The optimized SAM effectively suppressed these redundant detections caused by reflection artifacts, thereby improving the precision to 0.94, the highest among the evaluated models for condensation images.

In addition to precision, we also evaluated recall to assess the model's ability to capture all droplets present in high‐density condensation images. The optimized SAM achieved a recall of 0.72, which was higher than that of the default SAM (0.68) and Mask R‐CNN (0.18). The improvement indicates that the optimized SAM more effectively detected small and low‐contrast droplets that were frequently missed by the other models. This balance of high precision (0.94) and high recall (0.72) highlights the robustness of the proposed framework in reliably segmenting both large and microscale droplets under diverse experimental conditions. It should be noted, however, that the recall values are lower than precision, primarily due to the scale discrepancy between predictions and ground truth annotations. The model can only detect droplets with a minimum radius of 4 pixels (corresponding to ∼40 µm) in a CMOS camera, whereas the ground truth includes droplets even smaller than this size. Consequently, very fine droplets annotated in the ground truth but below the model's detection limit reduce the recall, even though the framework performs reliably within the resolvable size range.

### 2.3 Computer Vision Analysis for Droplet Condensation

#### 2.3.1 Microscale Condensation Dynamics

**FIGURE 3:**
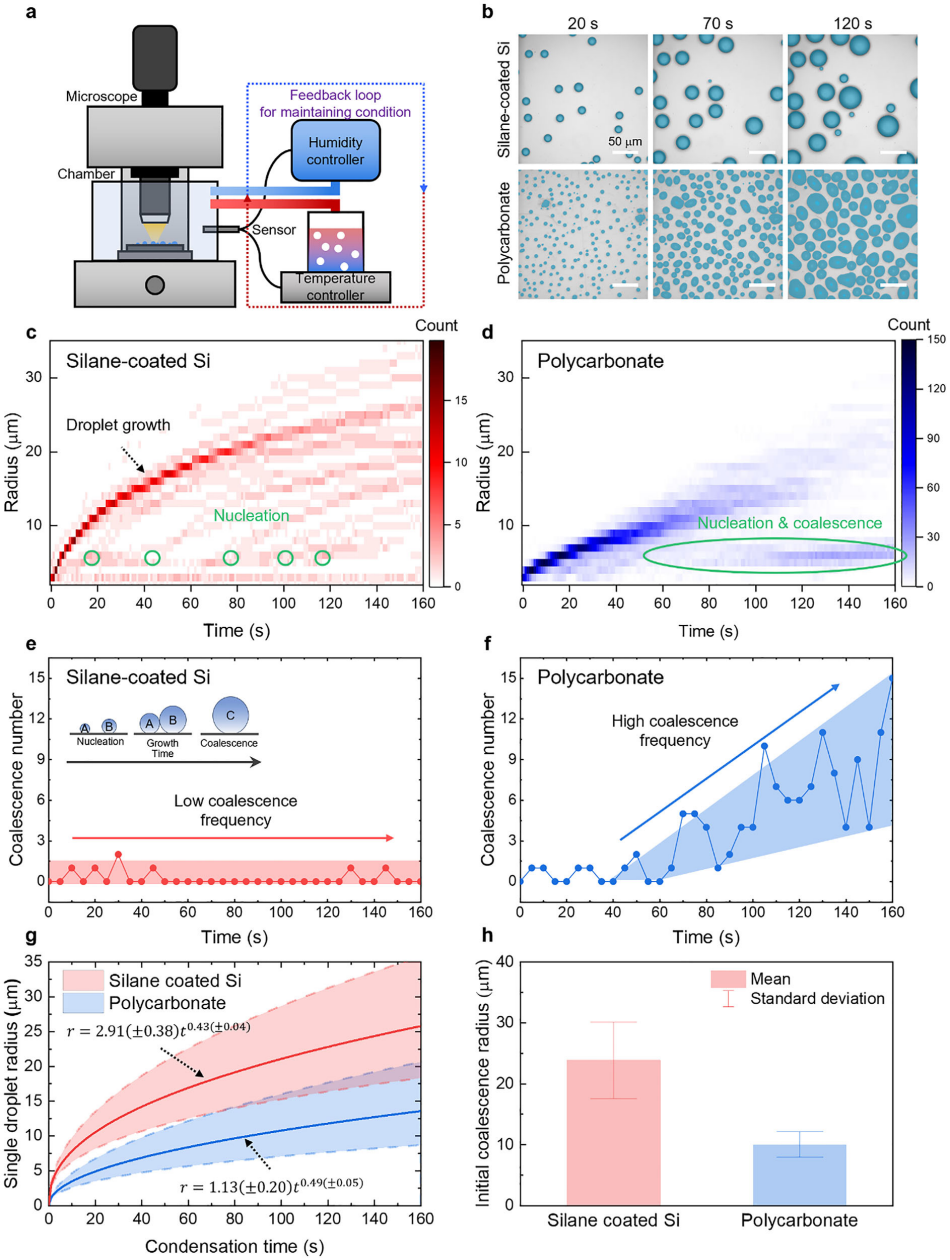
Microscale condensation dynamics analysis. (a) Experimental setup with controlled surface temperature, air temperature and humidity. (b) Time‐lapse optical images on silane‐coated silicon with sparse nucleation and polycarbonate with dense nucleation, captured with a field of view of 343 µm × 343 µm with a scale bar of 50 µm. (c,d) Temporal evolution of droplet size distribution. Silane‐coated silicon (red) exhibits narrower distribution and lower coalescence, but polycarbonate (blue) shows broader size dispersion due to frequent coalescence. (e,f) Coalescence frequency. Silane‐coated silicon (red) shows low coalescence frequency due to sparse nucleation site at initial, while polycarbonate (blue) exhibit increased coalescence after 40 s due to achieving coalescence radius. (g) Single droplet growth rate quantified by droplet tracking method. (h) A mean coalescence radius prior to first merge. Silane‐coated silicon exhibited an average radius of 23.9 ± 5.9 µm, compared with only 10.0 ± 2.0 µm on polycarbonate.

The condensation behaviors on silane‐coated silicon and polycarbonate surfaces were compared using optical microscopy (Figure 3). Figure 3a illustrates the experimental setup. The air temperature and relative humidity were controlled at 40°C and 80% ± 5%, respectively, using a feedback loop system composed of temperature and humidity controllers with integrated sensors, to ensure consistent environmental conditions. The sample surface temperature was maintained at 7°C using a cooling stage. This condition represents a high effective supersaturation level of *S*_eff_ = 5.9. To normalize the environmental condition across experiments, *S*_eff_ = *P*_vapor_ /*P*_sat_(*T*_s_) was used as the reference criterion in ambient conditions, where *P*_vapor_ is the vapor pressure, *P*_sat_(*T*_s_) is the saturation pressure at the surface temperature, and *T*_s_ is the sample surface temperature. Figure 3b shows time‐lapse condensation images of both samples, captured with a field of view of 343 µm × 343 µm with a scale bar of 50 µm. Here, *t*  =  0 s is defined as the first frame in which droplet nucleation becomes visibly observable within the field of view, and all subsequent times are measured relative to this condensation onset. Silane‐coated silicon surfaces exhibited significantly fewer droplets with larger sizes and lower eccentricity, indicating sparsely distributed nucleation sites. In contrast, the droplet characteristics on polycarbonate surfaces showed a higher density of smaller droplets, reflecting densely distributed nucleation sites and higher eccentricity due to high CAH (Table S1) [33]. This difference can be attributed to the disparity in surface roughness. The RMS roughness of silane‐coated silicon is 0.99 nm, whereas that of polycarbonate is 6.63 nm (Figure S4 and Table S3). According to classical nucleation theory, surface structures can lower the Gibbs free‐energy barrier for nucleation, and both molecular dynamics (MD) simulations and experiments have confirmed that rougher surfaces tend to exhibit higher nucleation densities. In addition, surface roughness contributes to CAH. On surfaces with high CAH, droplet pinning can occur during droplet growth or coalescence, leading to increased droplet eccentricity (details in Note S10) [34].

Figure 3c,d displays the temporal evolution of droplet size distribution on silane‐coated silicon and polycarbonate surfaces, respectively. On the silane‐coated silicon surface, the initial droplet growth is dominated by direct condensation, in which droplets grow by absorbing water molecules from the surrounding vapor. Coalescence is relatively infrequent because the distance between neighboring droplets is too large for merging, resulting in a relatively narrow size distribution (Figure 3c). The green circles indicate nucleation occurring in exposed substrate regions between existing droplets, and these droplets follow the same growth curve as earlier ones. In contrast, on the polycarbonate surface, the initial droplet growth is dominated by coalescence due to dense nucleation sites. The initial droplets form at nucleation sites by absorbing water molecules, but their growth is rapidly governed by frequent coalescence with adjacent droplets. As a result, the newly nucleated droplets are prone to immediate merging, leading to a broad and irregular size distribution dominated by coalescence‐driven dynamics rather than direct condensation growth (Figure 3d).

We quantified the droplet coalescence events using an individual droplet tracking method. Figure 3e,f presents the coalescence count by tracking individual droplets and counting the number of coalescence events per 5‐second interval. On the silane‐coated silicon surface (red), the coalescence count remains consistently low and nearly constant throughout the observation period, reflecting the sparse nucleation density and larger inter‐droplet spacing. In contrast, the polycarbonate surface (blue) exhibits a sharp rise in coalescence beginning at approximately 40 s, as the droplets grow larger and the inter‐droplet spacing decreases, promoting frequent merging once the droplets reach a critical radius. The pronounced difference between the two surfaces underscores the critical influence of surface roughness and nucleation site density on coalescence frequency and subsequent droplet growth dynamics.

Figure 3g illustrates the growth kinetics of individual droplets from nucleation until their first coalescence, with experimental data fitted to the classical power‐law model *r*  =  α*t* ^β^ [35, 36]. In these plots, the middle lines depict the average droplet radius, and the shaded regions indicate one standard deviation. For silane‐coated silicon, the fitted coefficients were *α* = 2.91 ± 0.38 and *β* = 0.43 ± 0.04, whereas polycarbonate yielded *α* = 1.13 ± 0.20 and *β* = 0.49 ± 0.05. These results indicate that droplets on silane‐coated silicon grow faster before coalescence, due to the wider inter‐droplet spacing that ensures more accessible vapor supply and minimizes diffusion field overlap, thereby sustaining direct condensation growth. In contrast, droplets on the polycarbonate surface exhibit a lower growth rate, which can be attributed to the high initial nucleation density that narrows the spacing between neighboring droplets and restricts the available vapor supply, leading to diffusion‐limited growth. Figure 3h compares the mean coalescence radius, measured from ten droplets immediately prior to merging. The average radius on silane‐coated silicon was 23.9 ± 5.9 µm, more than twice that on polycarbonate (10.0 ± 2.0 µm). This contrast reflects the markedly different nucleation site densities. According to the dropwise condensation model, the mean coalescence radius decreases as the nucleation site density increases ($r=1/4\sqrt{N_{s}}$) [37, 38, 39]. On the polycarbonate surface, the high nucleation density enforces close droplet spacing and leads to earlier merging at smaller radii, resulting in a smaller mean coalescence radius. In contrast, the lower nucleation density of silane‐coated silicon allows droplets to grow to substantially larger sizes before merging with neighbors, thus exhibiting a larger coalescence radius. Importantly, the mean coalescence radius serves as a critical parameter in condensation heat transfer models, as it defines the characteristic droplet scale that governs population statistics, surface renewal frequency, and heat flux, which collectively determine overall condensation performance. The ability of our framework to directly extract this parameter from experimental data provides a more reliable basis for predicting heat transfer performance.

#### 2.3.2 Macroscale Condensation Dynamics

**FIGURE 4:**
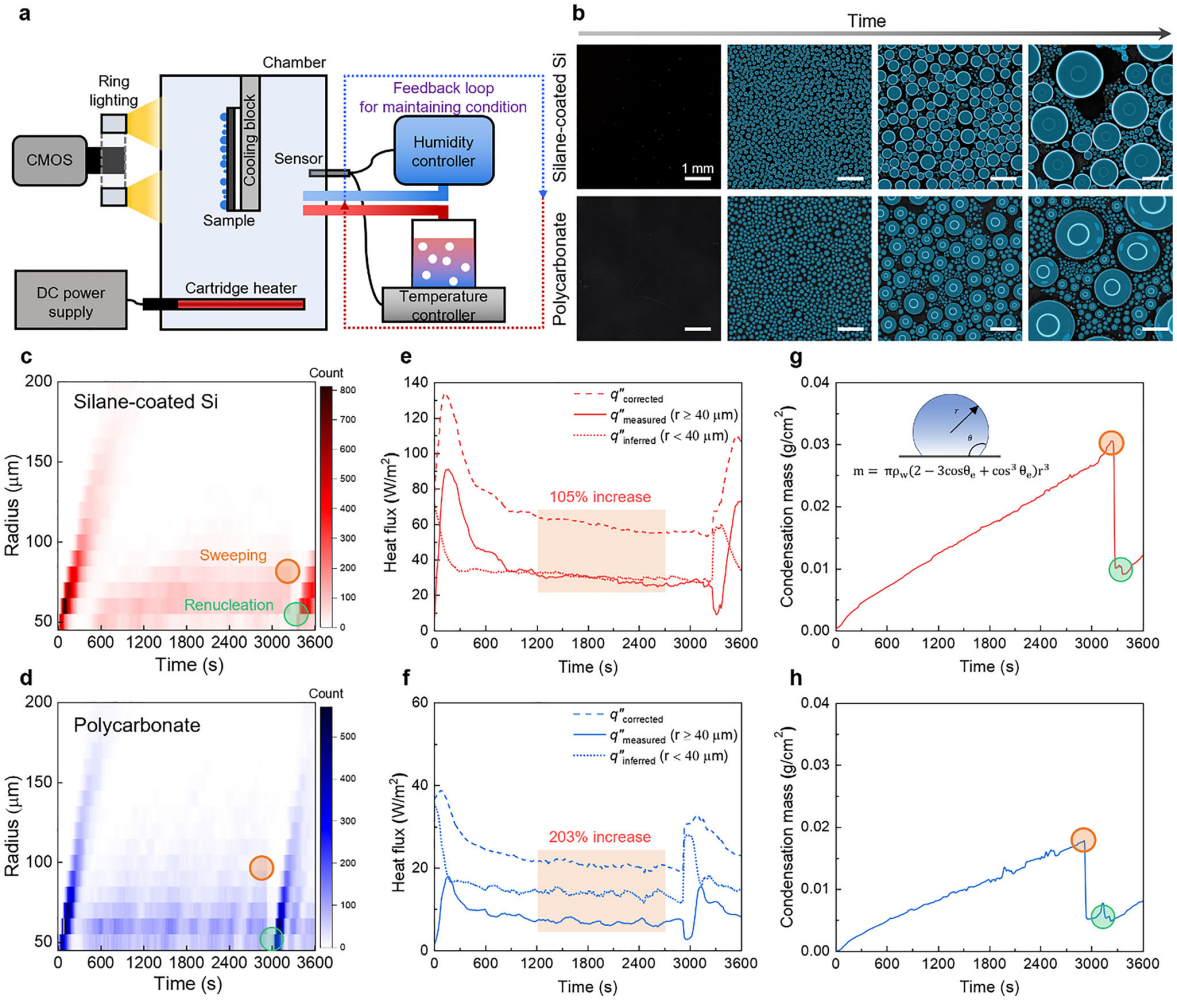
Macroscale condensation dynamics analysis. (a) Experimental setup with controlled surface temperature, air temperature and humidity for measuring droplets with a lower detection limit of 40 µm (see Figure S1 for a detailed description of the setup). (b) Optical time‐lapse sequences on silane‐coated silicon and polycarbonate. Scale bar in the image represents 1 mm. (c,d) Temporal evolution of droplet size distributions extracted using the computer vision framework. In these observations, droplet growth occurs mainly through coalescence with neighboring droplets. Orange circles indicate sweeping events, and green circles denote renucleation starting. (e,f) Spatially averaged heat flux calculated from droplet‐level features. Orange boxes indicate quasi‐steady regime between 1200 and 2800 s. (g,h) Cumulative condensation mass calculated by summing individual droplet volumes. Droplet mass increases linearly until sweeping (∼3200 s for silane‐coated silicon and ∼2900 s for polycarbonate), where large droplets depart and remove droplets along their path.

To compare the condensation performance of silane‐coated silicon and polycarbonate samples, several key features—including condensation mass, heat flux, droplet number, and size distribution—were extracted under controlled environmental conditions using the computer vision framework (Figure 4). Figure 4a shows the experimental setup where the ambient air temperature and relative humidity were maintained at 30°C and 75% ± 5% with a feedback control system, and the sample surface temperature was kept constant at 10°C using a cooling stage. These conditions correspond to an effective supersaturation level of *S*_eff_ = 2.6. Wide‐field images with a 5 mm × 5 mm field of view were obtained to capture macroscale condensation behavior. Figure 4b shows time‐lapse condensation images of both samples, providing a qualitative view of droplet evolution over the course of the experiment.

Figure 4c,d shows the temporal evolution of droplet size distributions on silane‐coated silicon (upper) and polycarbonate (lower) surfaces. For both samples the droplet number initially increases and reaches a peak around 100 s. As condensation proceeds, coalescence becomes dominant, which leads to a gradual decline in droplet number and the emergence of larger droplets in the distribution. Around 3000 s sweeping events abruptly reduce the droplet population as large droplets detach and remove neighboring droplets along their path. Following these sweeping events, renucleation occurs on the freshly exposed surface areas, resulting in a secondary increase in droplet number. This cyclic sequence of nucleation, growth, coalescence, sweeping, and renucleation is clearly captured in the histograms, demonstrating the ability of the computer vision framework to track large populations of droplets over extended periods.

In Figure 4e,f, the spatially averaged heat flux is presented, which was calculated from the droplet‐level physical features extracted through the computer vision framework. The heat flux associated with droplets that are directly resolved in the images acquired by the CMOS camera (minimum detectable radius ≈ 40 µm), denoted as 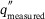 was determined by integrating the heat transfer rate of individual droplets on the surface and dividing by the surface area (details in Note S5). However, due to the spatial resolution limit of the CMOS system, droplets with radii smaller than 40 µm are not resolved in the images, even though such small droplets can contribute significantly to the actual heat flux. To compensate for this effect, we estimated the heat flux contributed by droplets with radii below 40 µm, denoted as 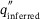, by combining the droplet‐free area fraction with the droplet number density. The corrected heat flux was defined as 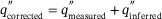 (details in Note S5).

In the corrected heat flux curves, both surfaces exhibit a pronounced early‐time peak at around 100 s, where the silane‐coated silicon and polycarbonate surfaces reach approximately 134 W/m^2^ and 39 W/m^2^, respectively. In this peak, the surface is densely covered with small droplets, which greatly enlarges the effective liquid–vapor interfacial area and thus maximizes the instantaneous heat transfer. As time progresses, coalescence drives the droplet population toward larger radii, and the associated increase in thermal resistance leads to a gradual decay in the heat flux. The higher peak heat flux observed on the silane‐coated silicon, compared with polycarbonate, is primarily attributed to droplet growth rate and effective nucleation sites. As discussed previously, the CMOS captures a regime in which the mean droplet radius has already exceeded the mean coalescence radius. In this regime, the droplet number and size distribution on the surface are governed more by coalescence, pinning, and droplet mobility. Compared with the silane‐coated silicon surface, the polycarbonate surface has a lower droplet growth rate, and its hydrophilic character combined with high CAH promotes pinning, resulting in distorted droplets with large projected areas, which reduces the droplet‐free area available for new nucleation and growth of resolvable droplets in the CMOS‐resolved regime. As a result, the silane‐coated silicon exhibits a higher heat flux than polycarbonate.

Furthermore, in the quasi‐steady regime between 1200 and 2800 s, comparison of 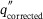 and 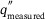 shows that the corrected heat flux increases on average by 105% for silane‐coated silicon and 202% for polycarbonate. This implies that, if droplets smaller than 40 µm are neglected, only about half of the actual heat flux is captured for silane‐coated silicon and roughly one third for polycarbonate. The larger discrepancy for polycarbonate is consistent with the much higher droplet number density in the 0–40 µm range compared with the silane‐coated silicon surface, as previously discussed in Section 2.3.1. Therefore, 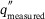 with radii ≥ 40 µm can be interpreted as a conservative lower bound on the true heat flux, whereas 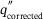 which includes the contribution from droplets smaller than 40 µm, provides an upper‐bound estimate under the present experimental conditions.

Figure 4g,h presents the cumulative condensation mass obtained by summing the individual droplet mass identified through the computer vision framework. For both samples, the condensation mass increased almost linearly until sweeping, reflecting continuous droplet nucleation and growth. The accumulation continued until sweeping events occurred at approximately 3200 s for silane‐coated silicon and 2900 s for polycarbonate, after which the cumulative mass abruptly decreased as departing large droplets swept across the surface, removing smaller droplets along their trajectories. By the time of sweeping, the silane‐coated silicon surface accumulated about 0.031 g of water, which was nearly 1.5 times higher than the 0.018 g measured for polycarbonate. This difference demonstrates that the lower contact angle hysteresis of silane‐coated silicon facilitates sustained droplet growth and more effective condensation capacity. In contrast, the higher hysteresis of polycarbonate leads to stronger pinning forces, which hinder droplet mobility and limit cumulative condensation despite its higher nucleation density.

#### 2.3.3 Statistical Analysis of Droplet Condensation

**FIGURE 5:**
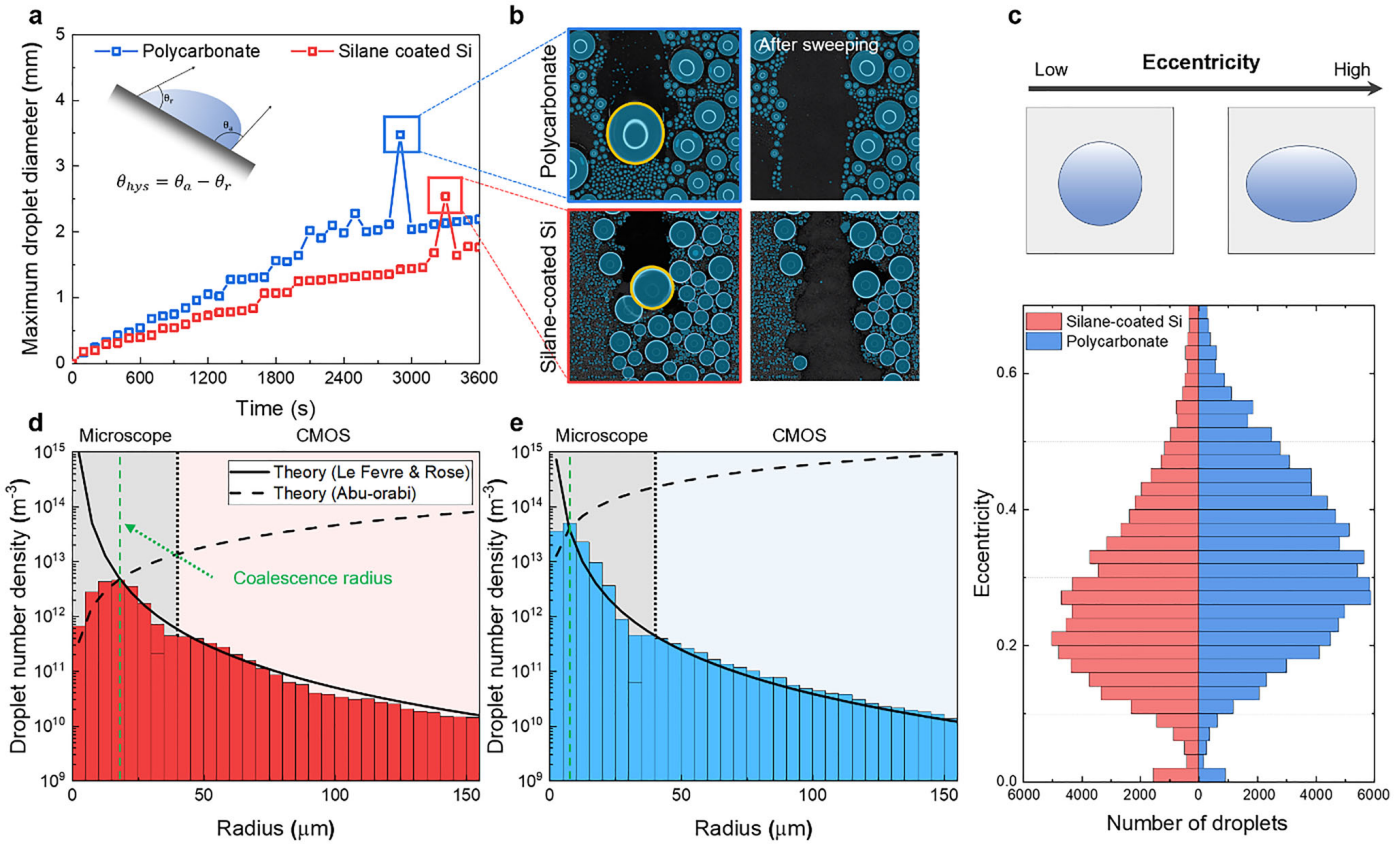
Statistical analysis of droplet condensation. (a) Maximum droplet diameters of silane coated silicon and polycarbonate from experimental results were measured in 3.5 and 2.5 mm, respectively. (b) Corresponding to condensation images before and after sweeping events, with departing droplets highlighted. (c) The distribution of droplet eccentricity, where polycarbonate (higher CAH, 30.0°) exhibits more deformed droplets compared to silane‐coated silicon (lower CAH, 11.6°). (d,e) Droplet number density for the silane‐coated silicon surface (d) and the polycarbonate surface (e). Solid lines in (d,e) indicate theory of Le Fevre & Rose, and dashed line denote theory of Abu–orabi. Intersection points both theory lines represent coalescence radius.

To investigate the role of contact angle hysteresis in governing condensation statistics, we measured the maximum droplet diameter (Figure 5a). From the initial nucleation, the maximum droplet diameter gradually increased by coalescence between neighboring droplets until the sweeping event. During coalescence‐mediated droplet growth, the maximum droplet diameter on the polycarbonate surface increased more rapidly than that on the silane‐coated silicon surface. This behavior may result from the surface characteristics of the polycarbonate, which has a higher nucleation site density and more frequent coalescence of larger droplets. Around 3000 s, a droplet exceeded the critical departure diameter, which the gravitational force overcomes the contact line pinning force, leading to spontaneous droplet departure and surface sweeping. As a result, the departure diameter was measured to be approximately 3.5 mm on the polycarbonate surface and 2.5 mm on the silane‐coated silicon surface. This difference is likely due to the larger contact angle hysteresis of the polycarbonate surface, which increases the pinning force. This sweeping behavior is directly visualized in the condensation images presented in Figure 5b.

To study the effect of contact angle hysteresis on the surfaces, we quantified the eccentricity of all individual droplets using the ellipse‐based metric defined as $e=\sqrt{1-{\left(b/a\right)}^{2}}\;$, where *a* and *b* denote the semi‐major and semi‐minor axes, respectively (Figure 5c). Eccentricity values close to 0 represent nearly circular droplets, whereas values near 1 indicate deformed shapes (upper panel). During condensation, we systematically analyzed the eccentricity over 150 000 droplets on each surface with the values discretized using a bin size of 0.02. As a result, the mean droplet eccentricity was 0.33 on the polycarbonate surface and 0.28 on the silane‐coated silicon surface. This is because the silane‐coated silicon surface exhibited lower hysteresis (CAH = 11.6°) compared to the polycarbonate surface (CAH = 30.0°), which is attributed to uniform surface roughness. These results demonstrate that the silane‐coated silicon surface produces more circular droplets due to reduced pinning.

To evaluate the droplet size distribution across the micro to macro scale, we combined the droplet number density data obtained from microscopy and CMOS imaging using the computer vision framework (Figure 5d,e). The values in the bar plot were discretized using a bin size of 5 µm. The dashed line represents the theoretical prediction based on the Abu–Orabi equation [40], which applies to droplets smaller than the coalescence radius, which was discussed in Section 2.3.1. Also, the solid line corresponds to the Le Fevre and Rose model [41], which is valid for droplets exceeding the coalescence radius (detail in Note S6). The reasonable agreement between experimental data and theoretical predictions validates the accuracy of the computer vision framework across different droplet size regimes. It demonstrates that droplet population statistics can be reliably extended from microscale nucleation dynamics to macroscale coalescence behavior. Due to its hydrophilicity, the polycarbonate surface exhibited a maximum nucleation density of up to 4.98 × 10^13^ droplets/m^2^ approximately an order of magnitude greater than that of the hydrophobic (4.53 × 10^12^ droplets/m^2^). This observation is consistent with classical nucleation theory.

### 2.4 Machine Learning‐Based Prediction of Condensation Rate

**FIGURE 6:**
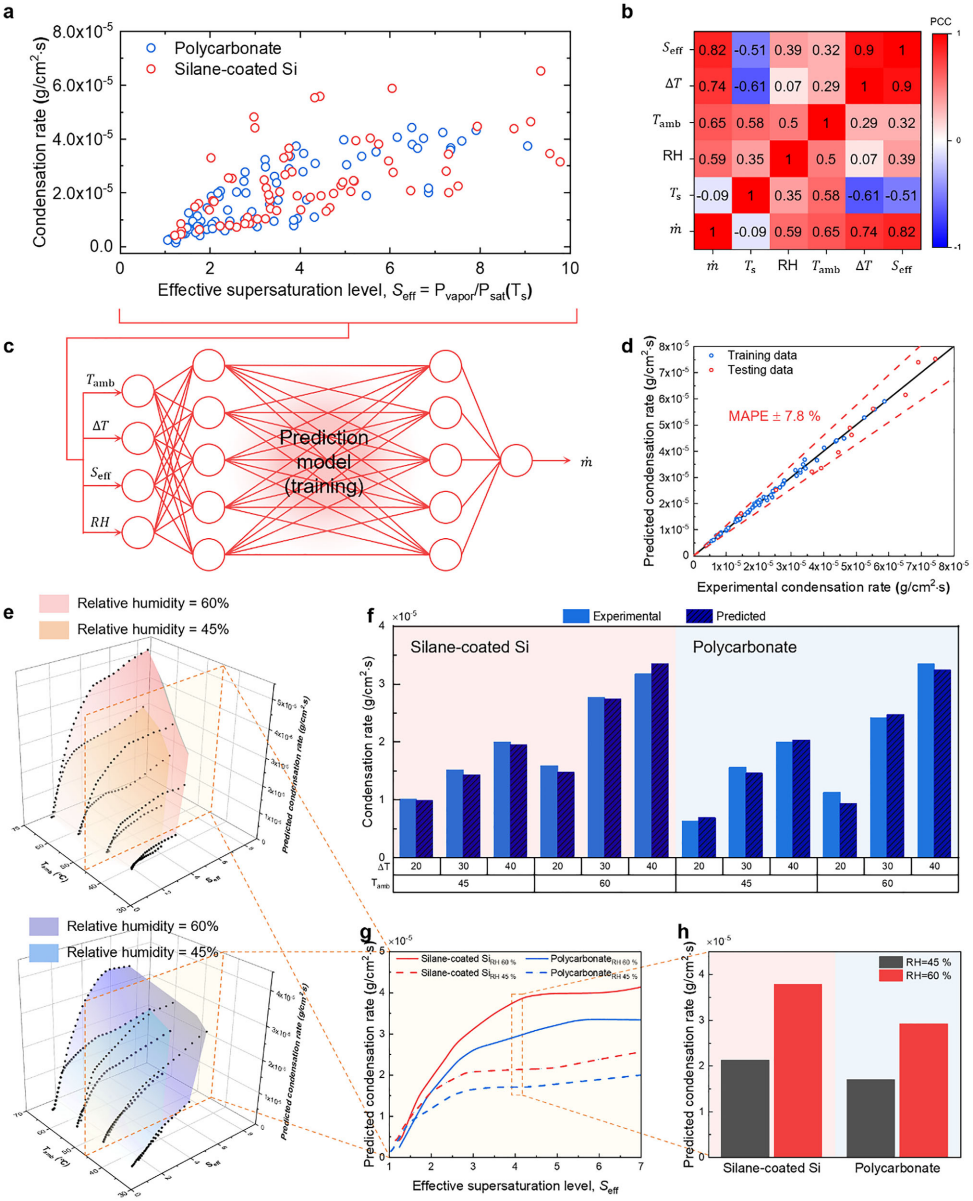
Machine learning‐based prediction of condensation rate. (a) The condensation rate datasets are obtained from experiments to train machine learning model. (b) The map of Pearson correlation coefficient results. (c) Machine learning model training process. The input parameters are determined as a result of Pearson correlation coefficient in (b). (d) The comparison between predicted condensation rate and experimental condensation rate. (e) Predicted 3D condensation rate surfaces for silane‐coated silicon (upper) and polycarbonate (lower) at relative humidity levels of 60% and 45%, showing higher rates at increased humidity. (f) The validation results of condensation rates with experimental and predicted data. (g,h) Predicted condensation rate as a function of effective supersaturation at an air temperature of 45°C, showing rapid increase at low supersaturation level.

To predict the condensation rate depending on environmental conditions, we developed machine learning surrogate models that capture the condensation rate as a 3D function of the environmental parameters (Figure 6). These processes consist of Pearson correlation coefficient (PCC) analysis, structure/hyperparameter optimization, and model training and validation. Figure 6a shows the training dataset obtained from condensation experiments conducted in a customized acrylic chamber under conditions ranging from 30%−70% relative humidity, 30°C−65°C ambient temperature, and 5°C−40°C surface temperature. The dataset consists of 150 data points, including 80 data from polycarbonate and 70 data from silane‐coated silicon. Then, to identify the variables that are most strongly correlated with the condensation rate, we computed the Pearson correlation coefficient for each candidate input and summarized the results in a correlation matrix (Figure 6b) [42]. The absolute value of the Pearson correlation coefficient approaching 1 indicates a stronger linear correlation. According to PCC results, variables with absolute PCC exceeding 0.5 were selected as input features for model training, yielding four variables—RH (relative humidity), *T*_amb_ (ambient temperature), Δ*T* (Δ*T*  = *T*_amb_  − *T*_s_), and *S*_eff_. Next, the machine learning model was trained to predict condensation rates under various environmental conditions (Figure 6c). In this model, a fully connected neural network with nonlinear activation functions was employed using the four PCC‐selected input features, so that the nonlinear dependencies between these variables and the condensation rate could be captured. The preceding PCC analysis thus served as a pre‐filter to reduce the candidate input space before training the network. We optimized the model parameters using epoch = 1000 and learning rate = 0.0035 (detailed description in Note S7 and Figure S5). The trained model exhibited a mean absolute percentage error (MAPE) test error of 7.8% (Figure 6d).

Figure 6e shows the predicted 3D surface forms of the surrogate models for silane‐coated silicon (upper) and polycarbonate (lower), respectively. The surface plots display the predicted condensation rate at a relative humidity of 45% and 60%. Figure 6f compares experimental and predicted condensation rates under different environmental conditions. For both silane‐coated silicon and polycarbonate, the model predictions closely follow the experimental results across a range of subcooling temperatures (Δ*T* = 20−40 K) and ambient temperatures (*T*_amb_ = 45°C and 60°C). The overall agreement confirms that the model reliably captures the influence of thermodynamic driving forces on condensation rate. In particular, the condensation rate increases systematically with both *T*_amb_ and Δ*T*, consistent with the enhanced supersaturation and stronger vapor‐to‐liquid driving force at higher thermal gradients. The close match validates the robustness of the model and establishes its potential for extrapolating condensation behavior to untested regimes.

Figure 6g,h shows the condensation rate predicted by the machine learning model as a function of *S*_eff_ at an ambient temperature of 45°C. For both samples, the condensation rate rapidly increases at lower supersaturation levels but tends to plateau as *S*_eff_ becomes very high. This trend is consistent with previous studies showing that while supersaturation enhances nucleation probability and the thermodynamic driving force for condensation, excessively high supersaturation does not proportionally accelerate the condensation process. At such conditions, the interfacial mass transfer is limited by the accommodation coefficient, which represents the probability that impinging vapor molecules are incorporated into the liquid phase, thereby introducing kinetic resistance to further growth. At an effective supersaturation level of 4 and a relative humidity of 60%, the predicted condensation rates for silane‐coated silicon and polycarbonate are 3.09 × 10^−5^ and 2.58 × 10^−5^ g/cm^2^ · s, respectively (Figure 6g).

## 3 Conclusion

In summary, we have established a label‐free computer vision framework for multiscale condensation analysis. Leveraging the zero‐shot capability of the Segment Anything Model (SAM), our approach accurately segmented more than one million droplets without annotated datasets, enabling robust extraction of droplet statistics such as radius, eccentricity, growth rate, condensation mass, and heat flux. Importantly, the framework directly quantified coalescence events and the mean coalescence radius, a critical parameter long assumed in classical models but rarely measured experimentally. By linking these droplet‐scale dynamics to macroscale performance, the method provides a stronger physical basis for condensation heat transfer predictions. In addition, a machine learning–based surrogate model was developed to predict condensation rates under diverse environmental conditions with high accuracy, reproducing both experimental measurements and physically consistent supersaturation trends. With its capability, scalability, and generality, this integrated framework offers a powerful tool for decoding condensation mechanisms and guiding the rational design of advanced thermal management, desalination, and water harvesting systems.

## 4 Experimental Section

### 4.1 Sample Preparation

A hydrophobic silicon surface was fabricated by functionalizing a polished silicon wafer with 1H,1H,2H,2H‐Perfluorodecyltrimethoxysilane (HTMS, CAS No. 83048‐65‐1, Sigma–Aldrich) through chemical vapor deposition (CVD) [43, 44]. The silicon wafer samples were cleaned in an ultrasonic bath with acetone, ethanol, and IPA for 5 mins and then dried using nitrogen gas. Afterward, the samples were treated with oxygen plasma for 10 min. For a hydrophobic surface, the samples were placed in a stainless steel container with a petri dish including 10 mL of toluene and 0.5 mL of HTMS. Then the container was heated at 80°C for 3 h. The contact angle of the hydrophobic silicon wafer surface was measured to be θ_**s**_ = 107.2° ± 0.6°, θ_**a**_ = 113.9° ± 1.0°, and θ_**r**_ = 102.3° ± 0.7°, as listed in Table S1.

The polycarbonate (PC) sample was obtained from Hyundai Mobis (samyang 3025U BK(H)‐3439K, South Korea). The contact angle of the polycarbonate surface was measured to be θ_s_ = 92.0° ± 2.3°, θ_a_ = 96.2° ± 1.9°, and θ_r_ = 66.2° ± 0.6°.

### 4.2 Water Vapor CMOS Condensation Experiment

The experiments were conducted in an acrylic chamber to regulate the internal environment with varying air temperature and humidity (Figure S1). The relative humidity inside the chamber was set from 20% to 90% ± 5% using a humidity control system (Nextron) which supplies a mixture of dry and humid air to the chamber. Water vapor at 100°C was supplied into the chamber using a heating mantle (DAIHAN Scientific) to achieve a hot and humid environment. The air temperature inside the chamber was controlled from 30°C to 70°C ± 3°C using a cartridge heater. Samples were vertically affixed onto a Peltier module using a carbon tape, and the module was connected to a water‐cooling block with a chiller maintained at 5°C.

Condensed droplet images on the samples were recorded at 1 fps using a high‐resolution camera (A7500CU35E, HuaRay iRAYPLE; sensor pixel size 3.45 µm) equipped with a Bi‐telecentric Lens (DTCM110‐48, SZ Vico) with 0.346× magnification, which corresponds to an effective pixel size of 3.45 µm / 0.346 ≈ 9.97 µm per pixel. To ensure robust segmentation of the circular droplet shape, only droplets with a minimum radius of 4 pixels were considered, corresponding to a minimum detectable droplet size of approximately 40 µm. This setup produced images of 2048 × 2048 pixels, equivalent to an area of approximately 20.4 mm × 20.4 mm.

### 4.3 Microscopic Condensation Experiment

The experiments were conducted in an acrylic chamber to regulate the internal environment with varying air temperature and humidity. For the microscopic tests, the chamber conditions were set to an air temperature of 40°C and a relative humidity of 80 ± 5%. Samples were mounted horizontally on a Peltier cooling module using a carbon tape and cooled through a water‐cooling block connected to a chiller maintained at 5°C, which stabilized the sample surface temperature at 7°C.

High‐resolution condensation measurements were performed using a high‐speed microscopy system consisting of a Phantom Miro M110 (Phantom) camera mounted on an Olympus BX53 microscope equipped with a 50× ultra–long working distance objective (M Plan Apo SL 50×, Mitutoyo) [45]. Images were acquired at a resolution of 768 × 768 pixels, and calibration using a scale bar yielded an effective spatial resolution of approximately 0.45 µm per pixel, enabling reliable detection of droplets down to about 2 µm in size.

## Funding

This work was supported by the Basic Science Research Program (RS‐2022‐NR070617) through the National Research Foundation of Korea (NRF) funded by the Ministry of Science and ICT.

## Conflicts of Interest

The authors declare no conflicts of interest.

## Supporting information




**Supporting File**: advs73720‐sup‐0001‐SuppMat.docx.

## Data Availability

The data that support the findings of this study are available from the corresponding author upon reasonable request.
