# Supplementary material for: Analysis of Multiscale Condensation Phenomena Using a Zero‐Shot Computer Vision Framework
Source: Adv Sci (Weinh). 2026 Jan 7;13(10):e21372. doi: 10.1002/advs.202521372 (PMC12915158; doi:10.1002/advs.202521372)
Supplement: Supplementary file 1 — Supporting File: advs73720‐sup‐0001‐SuppMat.docx. [file ADVS-13-e21372-s001.docx]

Supporting Information

Analysis of Multiscale Condensation Phenomena Using a Zero-Shot Computer Vision Framework

Donghyeong Lee, Seokwan Roh, Jaewoo Jeong, Kuk-Jin Yoon, Jungchul Lee, and Youngsuk Nam*

*ysnam1@kaist.ac.kr

**Contents**

1. Experimental setup (Note S1)

2. Random regions crop test (Note S2)

3. SAM option optimization process (Note S3)

4. AFM results (Note S4)

5. Data reduction (Note S5)

6. Droplet number density (Note S6)

7. Machine learning modeling process (Note S7)

8. Generalization Performance of the SAM (Note S8)

9. Evaluation metrics (Note S9)

10. Effects of surface roughness on dropwise condensation dynamics (Note S10)

11. Comparison of vision-derived heat flux with classical theoretical prediction (Note S11)

12. Experimental setup (Figure S1)

13. Random regions crop test (Figure S2)

14. SAM option optimization results (Figure S3)

15. Measurement of surface morphology using AFM (Figure S4)

16. Machine learning modeling process (Figure S5)

17. Qualitative robustness of droplet segmentation across diverse substrates and imaging conditions (Figure S6)

18. Comparison of vision-derived heat flux with classical theoretical prediction (Figure S7)

19. The water contact angle for polycarbonate and silane-coated silicon (Table S1)

20. The Segment Anything Model automatic mask generator options (default and optimized) (Table S2)

21. The RMS and mean roughness results on polycarbonate and silane-coated silicon surface using AFM (Table S3)

**Note 1**

**Experimental setup**


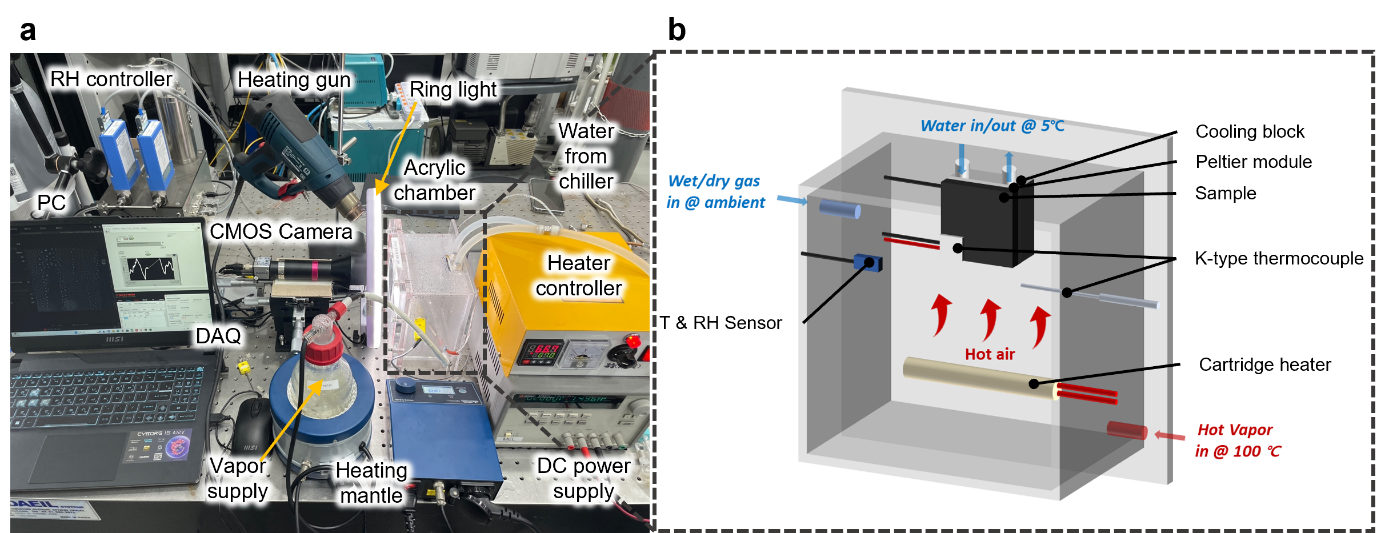


**Figure S1**. Experimental setup. a) Real image of the overall condensation experiments. All experiments were recorded using CMOS camera at 1 fps. The experimental data were acquired by a DAQ system and saved on a PC during condensation. b) Illustration of the experimental chamber. The chamber size was 100 mm × 100 mm × 150 mm. The temperature and humidity of the air during condensation were well controlled by the humidity controller and cartridge heater. The temperature of the sample was controlled by Peltier module connected with a cooling block with a water bath at 5 ℃, and the sample was mounted on the top surface of the Peltier module using carbon tape to ensure good thermal contact and reliable adhesion under hot and humid conditions. The temperature and humidity inside the chamber were monitored in real-time using high-precision temperature and humidity sensors and two K-type thermocouples.

**Table S1**. The water contact angle for polycarbonate and silane-coated silicon.

|  | **Advancing CA (°)** | **Static CA (°)** | **Receding CA (°)** | **CA Hysteresis (°)** | **Equilibrium CA (°)** |
| --- | --- | --- | --- | --- | --- |
| **Polycarbonate** | 96.2 ± 1.9° | 92.0 ± 2.3° | 66.2 ± 0.6° | 30.0 ± 2.0° | 81.5° |
| **Silane-coated Si** | 113.9 ± 1.0° | 107.2 ± 0.6° | 102.3 ± 0.7° | 11.6 ± 1.4° | 108.0° |

**Note 2**

**Random regions crop test**


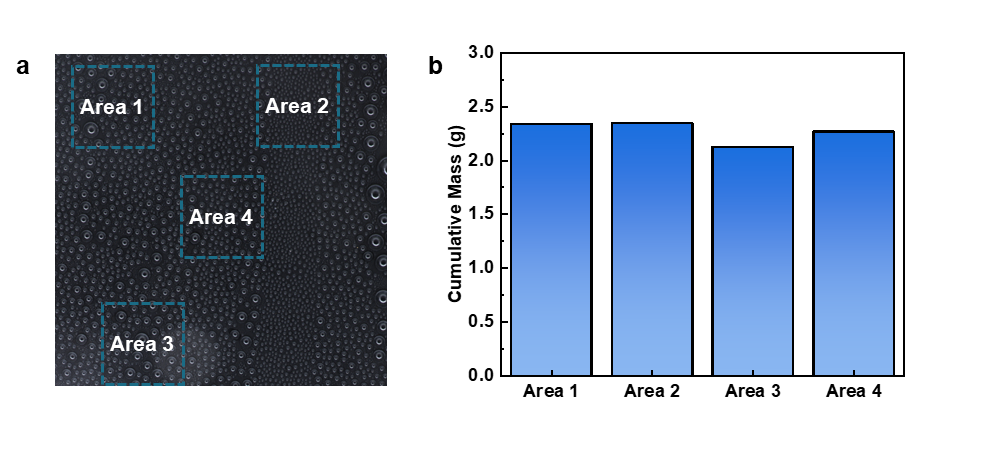


**Figure S2**. Random regions crop test a) Random crop test. The raw image (2048 × 2048 pixels) is cropped to 512 × 512 pixels to fit the model input requirements. b) The results of cumulative mass for random crop test.

In order to verify whether the condensation mass depends on the region, a random region crop test was conducted (Figure S2). In this test, four random areas from the original 2048 × 2048 pixels images were cropped to 512 × 512 pixels. For the four randomly selected regions, the cumulative condensed mass over one minute was 2.34, 2.35, 2.13, and 2.27 g for regions 1, 2, 3, and 4, respectively. These values exhibit an average difference of 3.3% and a maximum difference of 6.7% between regions. These results suggest that the condensation mass is independent of the selected region.

**Note 3**

**SAM option optimization process**


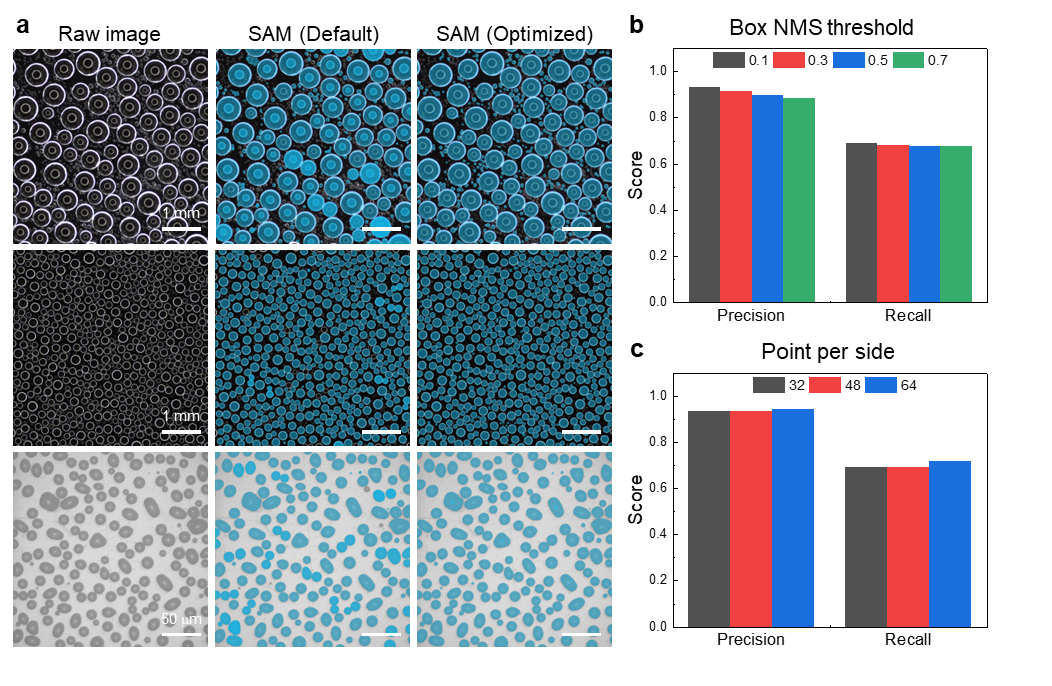


**Figure S3**. SAM option optimization results. a) The comparison with default SAM, and optimized SAM. Scale bars are provided within the images. b, c) SAM option optimization results. Box NMS threshold results (0.1, 0.3, 0.5, 0.7) and point per side (32, 48, 64).

**Table S2**. Segment Anything Model automatic mask generator options (default and optimized).

| **Mask generator option** | **Default** | **Optimized** |
| --- | --- | --- |
| Points per side | 32 | 64 |
| Points per batch | 32 | 256 |
| Predict IoU threshold | 0.88 | 0.88 |
| Stability score threshold | 0.95 | 0.95 |
| Box NMS threshold | 0.7 | 0.1 |

**Note 4**

**AFM results**


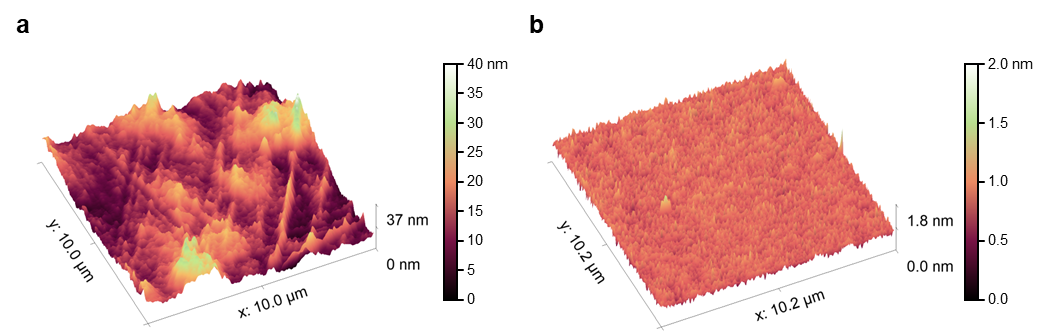


**Figure S4**. Measurement of surface morphology using AFM. a) Polycarbonate. b) Silane-coated silicon. The RMS and mean roughness are listed in Table S3.

**Table S3**. The RMS and mean roughness results on polycarbonate and silane-coated silicon surface using AFM.

|  | **RMS roughness** | **Mean roughness** |
| --- | --- | --- |
| **Polycarbonate** | 6.63 ± 2.61 nm | 4.82 ± 1.77 nm |
| **Silane-coated Si** | 0.99 ± 0.45 nm | 0.20 ± 0.05 nm |

**Note 5**

**Data reduction**

From the output of the vision framework, we obtained a set of information including droplet area, radius, the number of droplets, and location of individual droplets. These values should be converted into physically meaningful information, such as heat transfer rate, condensation mass, and droplet growth rate to extract significant insights.

The condensation heat transfer rate can be calculated as the product of the condensation rate and the latent heat of vaporization, based on the energy balance equation.^[1,2]^

| $q_{i}^{j}=\dot{m}_{i}^{j}h_{\mathrm{fg}}=\pi\rho_{w}h_{\mathrm{fg}}\left( 1-\cos\theta_{e} \right)^{2}\left( 2+\cos\theta_{e} \right)r_{i}^{j^{2}}\frac{dr_{i}^{j}}{dt}$ | (S1) |
| --- | --- |

where, $\dot{m}$ is condensation mass, $j$ represents the time index of the condensation images,$i$ represents the droplet index on the surface, $\rho_{w}$ is the density of water, $h_{\mathrm{fg}}$ is the latent heat of vaporization, $r$ is the radius of the individual droplet, $dr/dt$ is the growth rate of individual droplet, and $\theta_{e}$is the equilibrium contact angle, which is calculated by $\theta_{e}=\cos^{-1} (0.5\times\cos\theta_{a}+0.5\times\cos\theta_{r})$.

Heat flux at a specific time *j* can be calculated by summing the heat transfer rates of individual droplets and dividing by the area of sample surface.

| $q^{''j}=\frac{\sum_{i=0}^{n} q_{i}^{j}}{A_{s}}$ | (S2) |
| --- | --- |

where, *n* represents the total number of the droplets at a specific time *j* and $A_{s}$ is the area of the sample surface.

Droplet growth rate is used to obtain heat transfer rate. This is based on droplet growth followed by a power-law exponential model expressed as $r=\alpha t^{\beta}$*.*^[3-5]^

| $\frac{dr}{dt}=\beta\alpha t^{\beta-1}=\alpha^{\frac{1}{\beta}}\beta r^{\frac{\beta-1}{\beta}}$ | (S3) |
| --- | --- |

where, $\alpha$ and $\beta$ are the growth parameters, which can be obtained from fitting the experimental data.

In the CMOS-based condensation experiments, the effective pixel size is approximately 9.97 µm, and a minimum detectable droplet radius of 4 pixels was imposed in the image-processing pipeline. As a result, only droplets with radii r ≥ 40 µm are directly resolved in the CMOS images. The corresponding heat flux, denoted as $q_{\mathrm{measured}}^{''}$, is calculated by integrating the heat-transfer rate of all resolved droplets on the sample and dividing by the surface area, as described in Equation S2. However, droplets with radii below 40 µm are not resolved by the CMOS system, even though such small droplets can contribute significantly to the total heat transfer rate. To estimate their contribution, we combined the droplet area fraction obtained from the CMOS images with the droplet number density measured in the microscopic experiments.

For each time step *t*, the droplet area fraction measured from the CMOS images, $\phi_{\mathrm{measured}}(t)$, is defined as the ratio of the area covered by resolved droplets (r ≥ 40 µm) to the total image area. The complementary area fraction

| $\phi_{0-40 \mu m}(t)=1-\phi_{\mathrm{measured}}(t)$ | (S4) |
| --- | --- |

is interpreted as an effective area fraction occupied by sub-resolution droplets with radii in the range 0–40 µm.

The size distribution of these small droplets is reconstructed using the droplet number density $N_{d}(r)$ obtained from the microscopic measurements (Figure 5d and e). The microscopic data are binned with a radius increment of Δ*r* = 5 µm, so the interval 0–40 µm is discretized into eight bins with center radii

| $r_{i}=\left( i\boldsymbol{-}1/2 \right)\Delta r (i=1,2,\ldots,8; \Delta r=5 \mu m)$ | (S5) |
| --- | --- |

that is, $r_{i}=2.5, 7.5,\ldots,37.5 \mu m$. For each bin center $r_{i}$, the heat flux contribution of a single droplet, $q(r_{i})$, is evaluated using the energy-balance-based expression given in Equation S1, and multiplied by the corresponding droplet number density $N_{d}(r_{i}$) (per unit area and per bin size). The heat flux associated with droplets in the range 0–40 µm is then estimated as

| $q_{\mathrm{inferred}}^{''}=\phi_{0-40 \mu m}\sum_{i=1}^{8} q\left( r_{i} \right)N_{d}\left( r_{i} \right)\Delta r, \Delta r=5 \mu m$ | (S6) |
| --- | --- |

Here, $q(r_{i})$ is the heat flux contributed by a single droplet of radius $r_{i}$, $N_{d}(r)$ is the droplet number density from the microscopic experiments, and Δ*r* = 5 µm is the bin size. A discrete summation is used instead of a continuous integral because the microscopic number density data are provided in histogram form with a finite bin size. Finally, the corrected heat flux including both resolved and sub-resolution droplets is defined as

| $q_{\mathrm{corrected}}^{''}=q_{\mathrm{measured}}^{''}+q_{\mathrm{inferred}}^{''}$ | (S7) |
| --- | --- |

In the main text (Figure 4e and f), $q_{\mathrm{measured}}^{''}$ represents a conservative lower bound on the actual heat flux, because it neglects droplets smaller than 40 µm, whereas $q_{\mathrm{corrected}}^{''}$ provides an upper-bound estimate of the total heat flux that includes the inferred contribution from sub-resolution droplets under the present experimental conditions.

**Note 6**

**Droplet number density**

The distribution of droplet number density $N_{d}(r)$ was analyzed based on the theoretical framework described in Section 2.3.1, where the characteristic coalescence radius $r_{c}$ defines the mean coalescence radius.

By connecting the two regimes at $r=r_{c}$, the overall number density distribution can be written as a piecewise function:

| $N_{d}\left( r \right)=\left\{ \begin{aligned} n\left( r \right), r<r_{c} \\ N\left( r \right), r\geq r_{c} \end{aligned} \right.$ | (S8) |
| --- | --- |

where $n(r)$and $N(r)$ denote the droplet populations in the nucleation and coalescence regimes, respectively.^[6,7]^

| $n\left( r \right)=\frac{1}{3\pi}R_{f}^{-\frac{1}{3}}r_{e}^{-\frac{5}{3}-\frac{1}{\beta}}r^{\frac{1}{\beta}-1}$ | (S9) |
| --- | --- |
| $N\left( r \right)=\frac{1}{3\pi r^{2}R_{f}}\left( \frac{r}{R_{f}} \right)^{-\frac{2}{3}}$ | (S10) |

This unified description reproduces the experimentally observed distributions on both silane-coated silicon and polycarbonate surfaces.

The departure radius $R_{f}$ was determined from the force balance between gravitational and capillary adhesion forces acting on a droplet:

| $R_{f}=\sqrt{\frac{6\gamma\left( \cos\theta_{r}-\cos\theta_{a} \right)\sin\theta_{e}}{\pi\rho g(2-3\cos\theta_{e}+\cos^{3} \theta_{e})}}$ | (S11) |
| --- | --- |

where $\gamma$ is the surface tension of water, $\rho$ is the liquid density, $g$ is gravitational acceleration, and $\theta_{a}$, $\theta_{r}$, and $\theta_{e}$ denote the advancing, receding, and equilibrium contact angles, respectively.

This radius represents the critical droplet size at which the gravitational force overcomes the pinning force at the contact line, leading to spontaneous droplet departure (or sweeping). Experimentally, $R_{f}$ provides a quantitative threshold distinguishing attached and departing droplets, and it serves as a key parameter for evaluating macroscopic condensation efficiency and surface renewal frequency.

**Note 7**

**Machine learning modeling process**

**
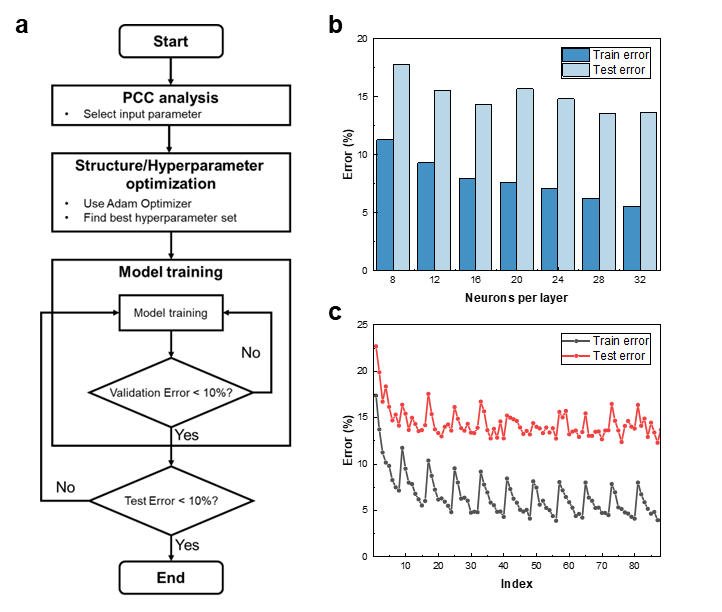
**

**Figure S5**. a) The processes of machine learning modeling. b) The results of structure optimization for machine learning model. c) The results of hyperparameter optimization for machine learning model.

We conducted structure and hyperparameter optimization for the machine learning modeling. For structure optimization, we evaluated the number of neurons per layer, starting from 8 and increasing by increments of 4 up to 32. As presented in Figure S5b, the selected structure with 32 neurons per layer achieved the lowest test error of 13.6%. Figure S5c shows the results of hyperparameter optimization, where the learning rate of the Adam optimizer ranged from 0.0005 to 0.0055 in increments of 0.0005 (11 cases), and the number of epochs ranged from 300 to 1000 in increments of 100 (8 cases), resulting in a total of 88 hyperparameter combinations.^[8]^ As a result, the 71st hyperparameter set with a learning rate of 0.0035 and 1000 epochs was selected due to its lowest test error. Based on the optimized model, training was performed. As a result, the best model achieved train and test errors of 2.5% and 9.8%, respectively.

**Note 8**

**Generalization Performance of the SAM**


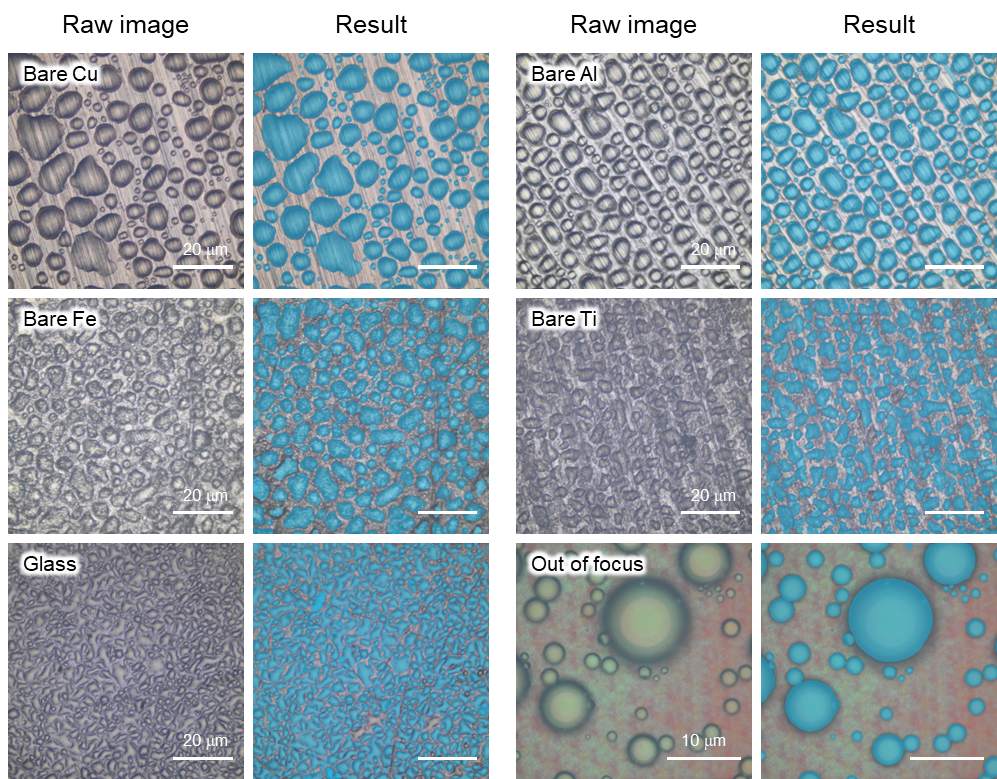


**Figure S6**. Qualitative robustness of droplet segmentation across diverse substrates and imaging conditions. Representative raw images (left) and corresponding segmentation image (right) acquired on bare Cu, Al, Fe, Ti, and glass, with an additional out-of-focus example on a nanostructured surface, demonstrating consistent droplet identification across substrate-dependent texture/contrast variations and focus-induced contour blurring. Scale bars are provided in each image.

**Note 9**

**Evaluation metrics**

To quantitatively evaluate the performance of the proposed framework, we compared the predicted droplet detections with manually annotated ground-truth bounding boxes on a representative test set of condensation images acquired on both silane-coated silicon and polycarbonate surfaces. Evaluation was performed on 50 condensation images containing approximately 23,000 manually annotated droplet bounding boxes under various environmental conditions. The dataset consisted of 5 microscopic images and 20 CMOS images for each substrate (silane-coated silicon and polycarbonate), resulting in a total of 50 images.

For each image, we computed the intersection-over-union (IoU) between every predicted box and every ground-truth box and performed one-to-one matching based on the highest IoU. If the maximum IoU between a predicted box and a ground-truth box exceeded 0.5, this pair was counted as a true positive (TP). Predicted boxes that were not matched to any ground-truth box with IoU > 0.5 were counted as false positives (FP), and ground-truth boxes that remained unmatched were counted as false negatives (FN).

The performance metrics used to evaluate the detection model were precision and recall.^[9]^ In general, precision is defined as the ratio of true positive instances to the sum of true positive and false positive instances, and represents how often the model’s positive predictions are correct:

| $Precision = \frac{\mathrm{TP}}{TP+FP}$ | (S12) |
| --- | --- |

In the context of condensation experiments, precision therefore indicates the fraction of detected droplets that actually correspond to true droplets in the ground-truth annotations.

Recall is defined as the ratio of true positive instances to the sum of true positive and false negative instances, and represents how often the model correctly retrieves positive instances among all positives present in the data:

| $\mathrm{Recall} = \frac{\mathrm{TP}}{TP+FN}$ | (S13) |
| --- | --- |

For our condensation experiments, recall thus quantifies the fraction of ground-truth droplets in the images that are successfully detected by the model.

These metrics were used to systematically assess the reliability of droplet detection across different substrates and imaging conditions.

**Note 10**

**Effects of surface roughness on dropwise condensation dynamics**

According to classical nucleation theory, condensation requires overcoming a Gibbs free-energy barrier.^[10]^ For heterogeneous nucleation, the energy barrier is lower than that for homogeneous nucleation, which is why condensation preferentially nucleates on surfaces. Similarly, geometrical structures can further reduce the energy barrier relative to a flat surface and thus serve as nucleation sites.^[11]^ In other words, a rougher surface can provide more nucleation sites, resulting in a higher droplet number density. This trend has been confirmed in molecular dynamics (MD) simulations that capture nucleation behavior,^[12]^ as well as in experiments using optical microscopy^[13]^ and environmental scanning electron microscopy (ESEM).^[14]^

Surface roughness is also one of the primary contributors to contact angle hysteresis (CAH).^[15]^ Surface defects can pin the droplet contact line, leading to energy dissipation.^[16]^ Therefore, under otherwise identical condensation conditions, higher surface roughness can hinder contact-line advancement and reduce the apparent droplet growth rate. Moreover, contact-line pinning can reduce the area available for re-nucleation, potentially decreasing the heat flux.^[17,18]^ In contrast, on samples with low CAH, the contact line is less likely to pin even after coalescence, allowing small droplets to nucleate in newly exposed regions. Accordingly, although the initial nucleation droplet density on PC is higher than that on silane-coated Si, the droplet density and heat flux on silane-coated Si can become higher than those on PC once droplet coalescence becomes active.

In addition, because the pinning force is larger on high CAH surfaces, droplets must grow to a larger size before gravity-driven sweeping (or sliding/departure) can occur.^[19]^ As a result, the high CAH PC surface exhibits a larger departure diameter than silane-coated Si, and droplet sweeping events are more frequent. Promoting more frequent removal of smaller droplets is a key strategy for enhancing dropwise condensation heat-transfer performance.^[20]^

**Note 11**

**Comparison of vision-derived heat flux with thermal network model** **prediction**

**Figure S7**. Comparison of vision-derived heat flux with theoretical thermal network model of Kim and Kim. Overall heat flux obtained from computer vision-based droplet statistics (Experiment) and from the Kim and Kim single-droplet model (Thermal network model) combined with the measured droplet population density $N(r)$ for silane-coated silicon and polycarbonate surfaces. The numerical values and detailed discussion are provided in Note S11.

To assess the consistency of the heat flux values obtained from computer vision-derived droplet statistics in Figure 4e and f, we compared the experimentally determined overall heat flux with a thermal network model based on the classical Kim and Kim single-droplet heat-transfer model.^[21]^ The thermal network model-based heat flux was computed by combining the Kim and Kim formulation for individual droplets with the experimentally measured droplet population density $N(r)$ (Figure S7).

For the silane-coated silicon surface, the experimentally obtained overall heat flux is 71 $W/m^{2}$, while the corresponding thermal network model prediction is 172 $W/m^{2}$, indicating that the experiment yields a value approximately 59% lower than the thermal network model estimate. Similarly, for the polycarbonate surface, the experimental and theoretical heat flux values are 24 $W/m^{2}$ and 58 $W/m^{2}$, respectively, again showing a reduction of about 59% relative to the idealized prediction.

This systematic difference is attributed to the influence of non-condensable gases (NCG) present in the moist-air environment of the experiments.^[22-24]^ Classical dropwise-condensation models, including that of Kim and Kim, typically assume pure-steam conditions or negligible NCG content.^[21]^ Under such assumptions, vapor transport to the droplet surface is limited only by interfacial thermal and mass-transfer resistances. In contrast, condensation in ambient air is governed by an additional vapor-side diffusion resistance, which increases the droplet-surface temperature, suppresses growth rates, and reduces the effective thermal driving force, $\Delta T_{\mathrm{dw}}=T_{\mathrm{dew}}-T_{w}$. Consequently, the area-averaged heat flux observed in experiments is expected to fall below the upper-bound predictions derived from pure-steam models.

Overall, this comparison confirms that the heat flux values computed from vision-based droplet statistics fall within the physically consistent range for moist-air condensation and are well aligned with the trends anticipated when NCG effects are considered.

**[References]**

1 Suh, Y., Lee, J., Simadiris, P. et al., "A deep learning perspective on dropwise condensation". *Adv. Sci.* **8**, 2101794 (2021).

2 Chen, L., Shi, D., Kang, X., Ma, C. & Zheng, Q., "Deep Learning Enabled Comprehensive Evaluation of Jumping-Droplet Condensation and Frosting". *ACS Applied Materials & Interfaces* **16**, 25473-25482 (2024). https://doi.org/10.1021/acsami.4c00976

3 Beysens, D., Steyer, A., Guenoun, P., Fritter, D. & Knobler, C., "How Does Dew Form?". *Phase Transitions: A Multinational Journal* **31**, 219-246 (1991). https://doi.org/10.1080/01411599108206932

4 Miljkovic, N., Enright, R. & Wang, E. N., "Effect of droplet morphology on growth dynamics and heat transfer during condensation on superhydrophobic nanostructured surfaces". *ACS nano* **6**, 1776-1785 (2012).

5 Chavan, S., Cha, H., Orejon, D. et al., "Heat Transfer through a Condensate Droplet on Hydrophobic and Nanostructured Superhydrophobic Surfaces". *Langmuir* **32**, 7774-7787 (2016). https://doi.org/10.1021/acs.langmuir.6b01903

6 Le Fevre, E. & Rose, J. W. in *International Heat Transfer Conference Digital Library.* (Begel House Inc.).

7 Abu-Orabi, M., "Modeling of heat transfer in dropwise condensation". *International journal of heat and mass transfer* **41**, 81-87 (1998).

8 Kingma, D. P., "Adam: A method for stochastic optimization". *arXiv preprint arXiv:1412.6980* (2014).

9 Powers, D. M., "Evaluation: from precision, recall and F-measure to ROC, informedness, markedness and correlation". *arXiv preprint arXiv:2010.16061* (2020).

10 Carey, V. P. *Liquid-vapor phase-change phenomena: an introduction to the thermophysics of vaporization and condensation processes in heat transfer equipment*. (CRC Press, 2020).

11 Aili, A., Ge, Q. & Zhang, T., "How nanostructures affect water droplet nucleation on superhydrophobic surfaces". *Journal of Heat Transfer* **139**, 112401 (2017).

12 Shi, Z., Zhong, S., Zhang, B., Wen, Z. & Chen, L., "Preferential water vapor condensation on a corrugated surface: A molecular dynamics study". *International Journal of Heat and Mass Transfer* **228**, 125623 (2024).

13 Koochak, P., Kiseleva, M. S., Lepikko, S. et al., "Smoothening Perfluoroalkylated Surfaces: Liquid‐Like Despite Molecular Rigidity?". *Advanced Materials Interfaces* **12**, 2400619 (2025).

14 Seo, D., Shim, J., Moon, B. et al., "Passive anti-flooding superhydrophobic surfaces". *ACS applied materials & interfaces* **12**, 4068-4080 (2019).

15 Butt, H.-J., Berger, R., De Coninck, J. & Tadmor, R., "Drop friction". *Nature Reviews Physics* **7**, 425-438 (2025).

16 Reyssat, M. & Quéré, D., "Contact angle hysteresis generated by strong dilute defects". *The Journal of Physical Chemistry B* **113**, 3906-3909 (2009).

17 Chu, F., Wu, X., Zhu, Y. & Yuan, Z., "Relationship between condensed droplet coalescence and surface wettability". *International Journal of Heat and Mass Transfer* **111**, 836-841 (2017).

18 Cha, H., Vahabi, H., Wu, A. et al., "Dropwise condensation on solid hydrophilic surfaces". *Science Advances* **6**, eaax0746 (2020). https://doi.org/doi:10.1126/sciadv.aax0746

19 Seo, D., Kim, Y., Seo, J. H. et al., "Modeling and optimization of hydrophobic surfaces for a two-phase closed thermosyphon". *International Journal of Heat and Mass Transfer* **165**, 120680 (2021).

20 Miljkovic, N., Enright, R. & Wang, E. N., "Modeling and optimization of superhydrophobic condensation". *Journal of Heat Transfer* **135**, 111004 (2013).

21 Kim, S. & Kim, K. J., "Dropwise Condensation Modeling Suitable for Superhydrophobic Surfaces". *Journal of Heat Transfer* **133** (2011). https://doi.org/10.1115/1.4003742

22 Ma, X.-H., Zhou, X.-D., Lan, Z., Li, Y.-M. & Zhang, Y., "Condensation heat transfer enhancement in the presence of non-condensable gas using the interfacial effect of dropwise condensation". *International Journal of Heat and Mass Transfer* **51**, 1728-1737 (2008). https://doi.org/https://doi.org/10.1016/j.ijheatmasstransfer.2007.07.021

23 Wang, J., Ma, Z., Li, G., Sundén, B. & Yan, J., "Improved modeling of heat transfer in dropwise condensation". *International Journal of Heat and Mass Transfer* **155**, 119719 (2020). https://doi.org/https://doi.org/10.1016/j.ijheatmasstransfer.2020.119719

24 Zheng, S., Eimann, F., Philipp, C., Fieback, T. & Gross, U., "Modeling of heat and mass transfer for dropwise condensation of moist air and the experimental validation". *International Journal of Heat and Mass Transfer* **120**, 879-894 (2018). https://doi.org/https://doi.org/10.1016/j.ijheatmasstransfer.2017.12.059
